# Perceptions about interventions to control schistosomiasis among the Lake Victoria island communities of Koome, Uganda

**DOI:** 10.1371/journal.pntd.0005982

**Published:** 2017-10-02

**Authors:** Richard E. Sanya, Edward Tumwesige, Alison M. Elliott, Janet Seeley

**Affiliations:** 1 Immunomodulation and Vaccines Programme, Medical Research Council/ Uganda Virus Research Institute (MRC/ UVRI) Uganda Research Unit, Uganda Virus Research Institute, Entebbe, Uganda; 2 Department of Internal Medicine, College of Health Sciences, Makerere University, Kampala, Uganda; 3 Social Aspects of Health Programme, Medical Research Council/ Uganda Virus Research Institute (MRC/ UVRI) Uganda Research Unit, Uganda Virus Research Institute, Entebbe, Uganda; 4 Department of Clinical Research, London School of Hygiene and Tropical Medicine, London, United Kingdom; 5 Department of Global Health and Development, London School of Hygiene and Tropical Medicine, London, United Kingdom; George Washington University School of Medicine and Health Sciences, UNITED STATES

## Abstract

**Background:**

Praziquantel-based mass treatment is the main approach to controlling schistosomiasis mansoni in endemic areas. Interventions such as provision and use of safe water, minimising contact with infested water, disposal of stool in latrines and snail control provide key avenues to break the transmission cycle and can sustain the benefits of mass treatment in the long term. Efforts are also being made to develop a schistosomiasis vaccine which, if effective, might reduce the incidence of re-infection after treatment. However, any interventions deployed need to be acceptable to, and sustainable by, the target communities.

**Methods:**

In this qualitative study, we investigated the perceptions of six Lake Victoria island communities of Koome, Uganda, about interventions to control *Schistosoma mansoni* infection and their willingness to participate in *Schistosoma* vaccine trials. Thirty-two in-depth interviews, 12 key informant interviews and 10 focus group discussions were conducted. Data were analysed using a thematic content approach.

**Findings:**

Intestinal schistosomiasis was not regarded as a serious health problem because a mass treatment programme is in place. However, the communities lack safe water sources and latrines. Mass treatment with praziquantel, safe water supplies and use of toilets were deemed the most acceptable interventions by the participants. The communities are willing to participate in *Schistosoma* vaccine trials.

**Conclusion/Significance:**

Knowledge of a community’s perception about interventions to control schistosomiasis can be valuable to policy makers and programme implementers intending to set up interventions co-managed by the community members. In this study, the views of the Lake Victoria island communities of Koome are presented. This study also provides data to guide further work on alternative interventions such as *Schistosoma* vaccine trials in these communities.

## Author summary

Schistosomiasis, a neglected tropical disease caused by the blood fluke *Schistosoma*, is still a huge burden in sub-Saharan Africa. The modalities for its control are mass treatment of the population with praziquantel, minimising contact with infested water, provision and use of safe water, intermediate host snail control and disposal of stool in toilets/latrines. For sustainable control of the parasite, the recipient communities need to embrace the interventions. In this study, we investigated the perceptions of fishing communities on the Lake Victoria Islands about interventions to control schistosomiasis and their willingness to participate in *Schistosoma* vaccine trials. We assessed their knowledge of schistosomiasis, their views on the interventions and the interventions most acceptable to them. We show that the community members of this schistosomiasis-endemic area prefer mass treatment with praziquantel, safe water supplies and use of toilets to minimise contact with infested water and snail control. The communities are also willing to participate in *Schistosoma* vaccine trials. This information is valuable to policy makers and programme implementers intending to set up interventions co-managed by the recipient communities. In addition, the study provides support for future *Schistosoma* vaccine trials in these communities.

## Introduction

Schistosomiasis affects an estimated 240 million people worldwide and over 90% of all *Schistosoma* infections are found in sub-Saharan Africa [1]. In Uganda, an estimated four million people are infected with *Schistosoma* and about 55% of the population is at risk of infection [2]. The Lake Victoria island communities of Koome sub-county Mukono district carry a large burden of intestinal schistosomiasis: in a recent study 52% of the inhabitants had *S*. *mansoni* infections detected on a single stool sample and over 70% using a rapid urine antigen test [3].

The morbidity caused by schistosomiasis is chronic and results in a huge socio-economic burden that is often underestimated [4]. The transmission cycle of intestinal schistosomiasis requires contamination of surface water by egg-laden human excreta, specific freshwater snails as intermediate hosts, and human water contact [5]. To break this cycle, existing intervention strategies include treatment with praziquantel, snail control, proper sanitation and provision of safe water supplies. For the interventions to be effective and sustainable, communities need to be provided with adequate health education [6–8].

Periodic mass treatment of communities with praziquantel is the most widely used approach to control schistosomiasis [9] with numerous gains reported [10, 11]. Although effective, it does have its drawbacks. Praziquantel does not kill immature schistosomes [12] and therefore does not clear all infection with single treatment, especially in individuals with high intensity infection [13, 14]. Since praziquantel does not prevent re-infection, the treatment must be provided repeatedly on a regular basis and the side effects can be unpleasant [15]. Due to these drawbacks and other factors such as poor drug coverage and poor drug compliance, control of schistosomiasis by mass treatment with praziquantel has not been consistent in the long-term [11, 16]. Therefore, effective long-term control of schistosomiasis by praziquantel mass treatment will rely on modifying the other components that facilitate infection transmission [17]. Without those additional interventions, re-infection rates will likely remain very high [18, 19]. However, a vaccine against *Schistosoma* could potentially overcome the challenges posed by mass treatment [17]; but currently none is commercially available [20]. Various antigens and vaccine candidates have been proposed [21] and some are investigated in clinical trials [22, 23] but none is yet readily approved and used for the public.

A long-lasting intervention against schistosomiasis needs to be cost-effective, acceptable to and sustainable by the recipient community. Therefore, gauging the readiness of communities in schistosomiasis endemic areas for intervention trials is of paramount importance[24]. In this paper, we investigated the perceptions of island communities with a high burden of the disease about their perceptions of schistosomiasis transmission and control. Specifically, we assessed their knowledge of schistosomiasis, their views on the various control strategies and the control interventions most acceptable to them. We also sought their opinion on their willingness to participate in *Schistosoma* vaccine trials. This knowledge can be valuable to policy makers and programme implementers intending to transition from project-provided interventions to interventions managed by the community members and local health facilities. This work also provides data to guide further work on alternative interventions such as *Schistosoma* vaccine trials in these communities.

## Methods

### Study setting and design

The study was carried out in the Lake Victoria island villages of Koome sub-county, Mukono district, Uganda. Mass drug administration of praziquantel is being provided to these communities as an intervention in a cluster randomised trial investigating the effects of anthelminthic intervention on health outcomes–the Lake Victoria Island Intervention Study on Worms and Allergy-related diseases (LaVIISWA) [3]–in collaboration with the Vector Control Division, Ministry of Health, Uganda. In this trial, 13 villages were randomised to receive the standard intervention against helminths (single dose praziquantel once a year, single dose albendazole twice a year) and 13 were randomised to intensive intervention (single dose praziquantel four times a year, triple dose albendazole four times a year). The interventions were rolled out in 2012 and are, in 2017, on-going.

The study presented here was cross-sectional and employed qualitative methods through in-depth Interviews (IDI), key informant interviews (KII) and focus group discussions (FGDs).

### Sampling

Six of the 27 fishing villages in the sub-county were randomly selected by the trial statistician to participate, taking into consideration that big and small villages were equally represented. Using STATA software (Stata Corp., College Station, TX, USA), a random selection of participating households in each village was generated.

Participants for the in-depth interviews were selected from six households in each village. Adult members of the household who had lived in Koome sub-county for at least 6 months were eligible to participate. On day 1, the selected households were contacted by the research team, a community leader and a member of the village health team. Eligible household members were invited to participate and appointments made to conduct the interviews during the week (Monday to Friday). One adult member was interviewed from each household, alternating between male and female and choosing the most “senior” adult available in each household (preferably the household head if this person was of the required gender). Six participants were interviewed per village (the first three male and three female participants to consent).

Two key informants were purposively selected per village. These were community leaders such as Local Council (LC) 1 chairpersons, Beach Management Unit (BMU) chairpersons, religious leaders and health workers. Beach Management Units are community fisheries management institutions set up in each fishing village.

Two focus group discussions were planned for each village, one for each gender (male and female). Five members were purposively selected for each group by the community leaders. These were community members aged 18 years and above and had lived in the sub-county for at least 6 months.

For each village, all the interviews (in-depth and key informant) and focus group discussions were conducted in a space of one week (Monday–Friday).

### Data collection

Prior to commencement of data collection, the study was presented to the district health team and consultations held with them. Thereafter, meetings were held in each of the six villages to present and explain the work to community members and answer questions about the study.

The data were collected by an experienced Social Science interviewer from the research team using both an audio recorder and field notes. Each interview lasted for about an hour. All the interviews and discussions were conducted in Luganda, the local language, using a translated topic guide (S4 Text, S5 Text, S6 Text). After the tools (information sheets, consent forms, interview topic guides and standard operating procedures) were developed, the procedures were piloted in one of the study villages. The key informant interviews were conducted in each village to obtain the views of the opinion leaders. The key informant interviews were conducted before focus group discussions. For each FGD, a moderator (from the research team) led the discussions, and a note-taker was present to document all verbal and nonverbal responses.

To assess awareness of the existence, causes, transmission, health problems and control of schistosomiasis, data were collected, using open-ended questions, on the following:

- The most important health issues in the community
- How bad the community thinks the problem of schistosomiasis is, compared to other major health issues
- Cause, modes of transmission of and health problems caused by schistosomiasis
- How to control schistosomiasis

The attitudes of the community members towards the following intervention strategies were assessed: mass administration of praziquantel, disposal of faeces in toilets or latrines, provision and use of safe water supplies, minimising contact with infested water and snail control.

To determine the schistosomiasis control interventions most acceptable for the community, views were solicited from the participants on the interventions they thought would work and were willing to adopt. Their opinions were also obtained on who should provide the interventions, how people can be motivated to use them and what the obstacles are.

The community members’ willingness to participate in future *Schistosoma mansoni* vaccine trials was also assessed. Characteristics of a mock vaccine trial were utilized and readiness assessed. The vaccine trial attributes considered were:

- Willingness to participate in a study and be available for a duration of up to 3 years
- Willingness to accept vaccine administration by injection
- Willingness to be randomised to any study arm including placebo
- Permission to have large volumes (8–10 teaspoons) of blood drawn
- Request to delay or defer pregnancy for 10 months (for women)

### Data analysis

All the notes and audio recordings were transcribed and the data were analysed manually using a thematic content approach. Responses were categorized into themes and ideas formulated by looking at the pattern of responses. After transcription, data were analysed thematically by closely reading and re-reading the interview scripts looking out for commonalities or recurring opinions and any other thoughts or ideas emerging from the data which formed our themes. The themes were then classified into subthemes and organised in relation to the study objectives. Narrative text was applied around the themes and participant direct quotes were added to illustrate the text.

The themes included assessment of community health problems, knowledge and awareness of schistosomiasis, perceptions about interventions to control schistosomiasis, knowledge about vaccines, and willingness to participate in *Schistosoma* vaccine trials.

### Ethics statement

Ethical approval to conduct this study was granted by the Research Ethics Committee of Uganda Virus Research Institute (reference number GC/127/15/05/510) (S1 Text), the London School of Hygiene and Tropical Medicine (reference number 10109) (S3 Text) and the Uganda National Council for Science and Technology (reference number SS 3831) (S2 Text). All participants provided written informed consent prior to the interviews and discussions.

## Results

Between October 2015 and January 2016, data were collected in the fishing villages of Kalyambuzi, Kachanga, Kisu, Kansambwe, Misenyi and Kisigala. Thirty-two in-depth interviews, 10 focus group discussions and 12 key informant interviews were conducted (94 participants interviewed in total) (see Supplementary material for tables from these three sources of data: S1 Table, S2 Table, S3 Table). In two villages, one FGD (instead of two) was conducted because of logistical constraints. The socio-demographic characteristics of the study participants are shown in Table 1.

**Table 1:** Socio-demographic characteristics of the study participants*.

	In-depth interviews (N = 32)	Key Informant interviews (N = 12)
Characteristics	Number (%)	Number (%)
**Sex**		
Female	17 (53)	05 (42)
Male	15 (47)	07 (58)
**Age**		
18–26	04 (12)	0
27–35	14 (44)	03 (25)
36–44	08 (25)	04 (33)
45+	06 19)	05 (42)
**Marital status**		
Married/ Co-habiting	24 (75)	10 (83)
Single	03 (09)	02 (17)
Widowed	02 (06)	0
Separated/ divorced	03 (09)	0
**Level of education**		
No formal education	03 (09)	0
Primary level	21 (66)	06 (50)
Secondary level	08 (25)	06 (50)
**Occupation**		
Fishing/ fishing related business	19 (59)	09 (75)
Farming	05 (16)	01 (08)
Other business (shop owner, bar etc)	06 (19)	02 (17)
Housewife	02 (09)	
Ownership of toilet/ latrine**		
Yes	07 (22)	06 (50)
No	25 (78)	06 (50)
Leadership position held in the community***		
LC 1 chairperson		02 (17)
BMU chairperson		03 (25)
Youth leader		01 (08)
Secretary LC 1		01 (08)
Women representative LC 1		05 (42)

*The characteristics of the individuals who participated in the FGDs were not recorded

**A toilet/ latrine was defined as a structure where a person may defecate and urinate

***The leadership positions of the individuals who participated in the IDIs were not recorded

### Major health issues in the community

A majority (63 out of 94) of the participants did not consider schistosomiasis to be a major health problem. To them, schistosomiasis had been a big problem in the past, which has been averted by mass drug administration of praziquantel.

*“It was bad in the past*. *In the morning when you went to the bush*, *you found people having passed stool with a lot of blood*, *diarrhoea was common*. *Today*, *yes I know that it is not good to defecate in the bush*, *at least when you go to the bush*, *you find that the stool passed is normal unlike in the past*. *Secondly*, *we no longer see people with distended abdomens ever since LaVIISWA started providing mass treatment*. *This was common among the fishermen especially those who fish tilapia since they frequently dive into the lake”*, 45 year old male, KII.

Now, schistosomiasis was not considered dangerous during its early stages and was regarded as not affecting activities of daily living. Respondents said that it only becomes more severe if left untreated and results in abdominal distension, body weakness, loss of appetite and eventually death.

*“Schistosomiasis is a very dangerous disease that can even kill if not treated*. *The stomach is the engine of the body and once it is affected*, *you can die*. *Yes*, *other diseases are also dangerous but you can easily know that you have them and go for treatment*, *which is also quite accessible*. *That is not the case for schistosomiasis”*, 45 year old male, KII.

The scarcity of toilets, safe water sources and health facilities were frequently reported as major health issues. The most frequently mentioned diseases affecting the community were malaria (mentioned by 32 participants), diarrhoea (27 participants), respiratory infections (28 participants) and HIV (25 participants).

### Knowledge about and sources of infection with schistosomiasis

Most of the participants had previously heard about schistosomiasis from community health workers and community leaders. A few said they studied about schistosomiasis at school.

*“I heard about schistosomiasis when I was still at school*. *I learnt about the causes and how it is contracted from infested water*. *When I came here in 2005 I saw a person suffering from schistosomiasis for the first time and he had a distended belly”*, Male participant, FGD.

However, despite having heard about it, all participants from the in-depth interviews, 7 out of 12 key informants and 33 out of 50 FGD participants reported, incorrectly, that the main source of infection with schistosomiasis was drinking infested water. Fifty-seven participants also correctly stated that contact with infested lake water (while fishing, fetching and washing from the lake, swimming and playing in the lake) and open defecation were sources of infection. Three participants said that eating half-cooked food or food that has been contaminated by flies which have come into contact with faeces causes schistosomiasis.

### Individuals most at risk of infection with schistosomiasis

Although everyone who lives or works in the study villages was perceived to be at risk of schistosomiasis infection, fishermen were identified as the most at risk. The other groups of people identified to be at risk are women who do laundry from the lake, children who swim in the lake for recreation and the youth who load and off load passenger boats.

### Control of schistosomiasis

Most (63 out of 94) participants stated that it is very hard to control schistosomiasis in their communities. They attributed this to the nature of their activities which revolve around the lake. Six key informants blamed this on the movement of people from one village to another, and inability of the community members to utilise the control measures in place.

*“It is very hard to prevent schistosomiasis as long as you live on the islands because it is very hard to avoid stepping in the lake*. *Most of our work requires coming into contact with the water”*, 40 year-old male, KII.

The control measures suggested by a few participants include improving sanitation, having access to safe and adequate water facilities, increasing the coverage of mass treatment and health education. Having a vaccine was also suggested as one of the interventions.

*“I don’t know if what I am going to say is achievable*, *just like they vaccinate measles and other diseases*, *vaccinating against schistosomiasis would help us a lot because all of us who stay here are always in contact with the lake”* Female participant, FGD.

One female participant reported placing water for domestic use in the sun for seven hours as a method she uses to prevent infection.

*“There is a belief in the community that once water is fetched and left out in the sun for about seven hours it becomes free from schistosomiasis and I have adopted this method*. *When you fetch it and you use it immediately*, *the chances of infection are high*, *even through the private parts while bathing”* 36 year old female, community leader.

Two female participants stated that their husbands draw water for domestic use from the middle of the lake as a measure to control schistosomiasis. They believed that water in the middle of the lake is unsuitable for the organisms to survive because it is warm. They also said they draw water for domestic use from routes used by ferries and other boats with big engines because they believe the organisms are repelled by the engines.

The treatment seeking behaviour of the community members was said to still be poor. Despite the free mass drug administration of praziquantel, some community members are said to be unwilling to accept treatment. One reason respondents cited for not taking treatment was being “too busy with their work”. It was also revealed that residents are reluctant to seek treatment for schistosomiasis during its early stages. This is because, they said, at these stages, they are asymptomatic and their daily activities are unaffected. Lack of health facilities in the sub-county for testing and treating schistosomiasis was also highlighted as a challenge.

### Perceptions about mass administration of praziquantel

Mass administration of praziquantel was perceived to be beneficial by 90 of the 94 participants. The fear of side effects notably dizziness, vomiting, fatigue, diarrhoea and loss of appetite was reported by a few participants as the reason for people’s refusal to take praziquantel. Men, especially fishermen, were reported to be the most notorious for dodging mass treatment for this reason. The side effects were described by one participant as being more severe than the disease itself. Most (56 out of 94) of the participants said the presence of side effects was because of infection with schistosomiasis. They said that the side effects show that the treatment is effective and these effects are short lived, while the benefits last longer. One participant complained about the bitter taste of the treatment tablets.

### Perceptions about disposal of faeces in toilets and latrines

Most participants (79 out of 94) viewed disposal of faeces in toilets as a good intervention to control schistosomiasis. They reported that disposal of faeces in latrines/toilets also helps to control the spread of other diseases like cholera, diarrhoea, dysentery and other worm infections. They noted that they lack latrines/toilets in their communities and this has resulted in open defecation. The major reason given for not owning a latrine was high cost of materials required for construction. In addition, it was said that the close proximity to the lake renders the soils too weak to keep a latrine firm. Eight participants blamed their landlords for the failure to have toilets in place.

*“The problem we have is that this land does not belong to us*. *We are tenants and our landlords don’t allow us to have toilets for our families*. *A piece of land covered by a toilet is a loss to the landlord because that same piece of land would be occupied by another person paying rent*. *That is the reason they don’t allow toilets here”*, 28 year old female, IDI.

Both the community members and the opinion leaders said some traditional beliefs deter people from owning and using toilets.

*“Some people have beliefs that they should not use toilets*. *They believe that they will not get fish or that they will not give birth when they use toilets*. *They always go to the bush and this continues to cause schistosomiasis”*, 26 year old male, KII.

However, one participant said negligence is the only reason why they do not have toilets. She said people in the islands believe that they are always on the move (temporarily settled) and so labouring to have toilets in place would be a waste of time and money.

*“……people here don’t mind*. *For a person to contribute shs1000 for a community toilet is not easy*. *He would rather buy alcohol and go to the bush or lake”*, Female, FGD.

### Perceptions about provision and use of safe water supplies

Nearly all participants (90 out of 94) perceived the provision of safe water supplies to be an effective intervention to control schistosomiasis. They reported that this intervention would mostly favour the women, children and people whose work does not involve regular contact with the lake such as bar and shop owners.

*“Safe water sources can only help to control schistosomiasis in some people like women and children*. *Most of us are fishermen and work on the lake therefore*, *even though safe water is provided*, *we will still get the disease from the lake”*, Male participant, FGD.

For this intervention to work more effectively, participants suggested that many safe water facilities must be erected. This will minimise long queues when accessing safe water and make fetching water directly from the lake less appealing.

### Perceptions about use of biological agents and chemicals to control snails

Most of the participants had reservations about the use of biological agents to control snails. Thirty-six participants felt that chemicals may affect the fish negatively because they (the chemicals) could be non-selective and kill the fish as well as snails. Two participants were keen to know the dose of the chemical required to kill all the snails. They also felt that this chemical may remain on the shores and not reach farther into the lake rendering it less effective.

*“There is no way that chemical will only kill snails and leave the fish*. *We have seen people using chemicals for fishing and as a result*, *snails are also killed*. *So anything that kills fish*, *kills snails and vice versa”*, Male participant, FGD.

On the use of biological agents such as fresh water prawns [25, 26] or ducks [27] to feed on the host snails, the participants were concerned about the numbers required. They expressed the fear that the agents (especially ducks) may be poached. Participants said that in the past, there were many wild ducks on Lake Victoria but now, they are almost extinct. On the use of competitor snails [28, 29] to control schistosomiasis, most (70 out of 94) participants wondered how snails can be predators of other snails. Some of the community leaders questioned the effectiveness of this intervention. Six participants who showed interest in the biological agents called for community engagement so that people can appreciate the potential of the intervention.

### Perceptions about minimising contact with infested water

There were mixed reactions about this intervention. The majority of female participants perceived it to be a good intervention saying that it is practical, provided there are other water sources in place. The male participants felt that this intervention is *‘*unrealistic’ and ‘unwanted’ because it will directly affect their income.

*“… (Laughs) that is very hard*. *Fishing is the source of our livelihood*. *None of us here came here for pleasure*, *we came to look for money*, *and our eyes are on that lake for a living*. *It is very hard to minimise contact with the lake*. *I will go and fish in the morning and if I get no fish and I will try again in the afternoon*. *If I still fail*, *I will go back in the evening*. *So it is quite hard”* Male participant, FGD.

Another participant from the female FGD said:

“That is not easy; children will always escape from their parents and go to the lake to swim. There is no way you can stop children. Even for us adults, there is when you feel hot and you go for a swim in the lake. We also go there to fetch water, to wash clothes. Therefore, I do not think minimising contact is possible when you still live here. Unless the whole lake is fenced and a gate put in place, there is no way”.

### Schistosomiasis control interventions considered most acceptable by the community

Most participants (51 out of 94) mentioned mass drug administration of praziquantel as an acceptable intervention which they are willing to adopt. This was closely followed by the provision of safe water sources (35 participants) and improved sanitation through disposal of faeces in toilets and latrines (30 participants). They said that the residents need to be educated on the proper disposal of faeces in order to curb open defecation.

Most participants felt that putting up such interventions requires extensive infrastructure which they are unable to provide because of poverty. Forty-four participants (47%) said the government should provide these interventions. Some (25 participants) suggested LaVIISWA should provide, and the rest suggested a joint venture between government and LaVIISWA or between government and non-governmental organisations (NGOs) or well-wishers.

Twelve participants (including three key informants) were unhappy with the way government operates. They said that government has on numerous occasions pledged to provide them with safe water and toilets. These pledges are yet to be fulfilled.

To sustain these interventions, participants felt that, once stringent penalties were in place, fines should be levied on those who do not comply. A few participants called for public engagement and health education. They said that community members should be told the advantages and disadvantages of the interventions before any penalties are enforced. Some participants said providing free access to the interventions will motivate people to use them.

*“…people will be motivated only when they don’t have to pay money to use them*. *If*, *for example*, *you charge a fee to use toilets*, *people will still go to the bush and Bilharzia will continue to be spread”*, 29 year old female, IDI.

### Willingness to participate in *Schistosoma* vaccine trials

Participants demonstrated that they had a basic knowledge about vaccines and their role in disease prevention. Many participants acknowledged that they were unaware of the availability of any *Schistosoma* vaccine. One participant thought that mass treatment with praziquantel was a form of vaccination. All participants said they would welcome a vaccine becoming available. The community leaders also reported that a vaccine would be the best intervention for controlling schistosomiasis.

*“I would feel so happy*. *Some of us knew how to swim but ever since we were told that schistosomiasis is contracted from the lake*, *we have since stopped*. *So with the discovery of a vaccine I would know I am going to revive my swimming skills”* Male participant, FGD.

*“That (vaccine) is even the most important one because many people fear tablets*, *but for a vaccine I am very sure all the residents in the camp would come out for vaccination”*, 40 year old male, KII.

The majority (74 out 94) of the participants expressed willingness to participate in a *Schistosoma* vaccine trial. They stated various reasons for the interest in enrolment: service to humanity, benefit from preventing schistosomiasis and trust in the researchers conducting the trial. The reasons for not participating were religious beliefs, conspiracy theories and fear of side effects.

The participants were willing to participate in a vaccine trial for a duration of three years although three participants felt that duration was too long.

The majority of the participants were willing to accept vaccine administration by injection. Seven participants (six of them were female) said they feared the pain caused by injections and would not participate in the trial for that reason. Most participants had no problems with being randomised to any study arm (including placebo). Eight participants preferred the arm with the candidate vaccine and gave it as a pre-condition for participating in the trial. Most participants said that they were willing to provide the required volumes of blood. Eight participants complained about the volumes and said they would not participate. Eleven participants said they should be given food supplements to replace the blood drawn and be provided with medical treatment during the trial. All but three female participants were willing to delay or defer pregnancy during the trial using birth control methods such as oral and injectable contraceptives. Five participants (all male) said they should be acknowledged as heroes and given some monetary compensation at the end of the vaccine trial.

## Discussion

In this paper, we have shown that the inhabitants of this schistosomiasis-endemic area prefer mass treatment with praziquantel, safe water supplies and use of toilets to minimising contact with infested water and snail control as the interventions they are willing to embrace. Despite awareness about the existence of schistosomiasis in their communities, they do not consider it as a major health priority because of a mass treatment programme in place. Gaps exist in their basic knowledge about schistosomiasis transmission and prevention such as regarding drinking of infested water as the main source of infection.

Provision of mass treatment with praziquantel has faced obstacles such as inadequate supplies of praziquantel, the costs associated with delivery to the target communities and lack of compliance with treatment [30]. Factors such as population migration, change in food supply, conspiracy theories about the intentions of MDA, fear of drug side effects and relations between drug distributors and the target community have been identified to determine MDA success in communities in Uganda [31]. MDA is also not taken up because of inappropriate and inadequate health education and differing biomedical and local understanding of schistosomiasis, absence during drug distribution, pregnancy, breast feeding and feeling healthy [32, 33]. Indeed the uptake of mass treatment with praziquantel has been sub-optimal in the Lake Victoria island communities [34] and other nearby communities [35]. With support from development partners such as the Schistosomiasis Control Initiative, praziquantel has become more affordable and the supplies more consistent. Logistical support and a motivated drug distribution network in these communities would ensure that the communities access the medication. Health education would address compliance.

Providing safe water supplies to the Lake Victoria island communities is still a challenge. Once achieved, the communities need to be educated on the benefits in order to maximise its use. Water for domestic use could be obtained from these safe sources and in the process, vulnerable groups such as women working at home and children would have less contact with the infested lake water. It will still be challenging to stop recreational contact and harder to convince the fishermen to minimise contact with the lake but attempts have to be made. In such a resource limited setting, a concerted effort involving the local communities, government and development partners is required to establish and sustain this intervention.

Despite the willingness to use toilets, coverage is very low (<10%) in these communities [3]. The communities feel that it is costly to construct and maintain latrines due to the terrain and some landlords are unwilling to provide land. Health education is also key in addressing the misconceptions about the source of infection and wrong beliefs about toilet use.

Despite mass treatment with praziquantel, safe water supplies and use of toilets being the most acceptable interventions, the communities felt that they are unable set up and sustain the interventions on their own. Reasons such as prohibitive costs in setting up and maintaining the interventions, mobility of the population, lack of unity in the communities owing to the diverse cultural backgrounds and uncooperative landlords were stated. The communities are willing to participate in sustaining the interventions and they provided suggestions such as setting up stringent byelaws, the need for health education and community engagement. Indeed, health education is important because knowledge about the transmission, severity and consequence of schistosomiasis may be poor [36]. As demonstrated elsewhere, the communities need to be involved in designing the interventions in order to promote ownership of the intervention [24].

To our knowledge, this is the first qualitative study to assess the willingness of a highly endemic community to take part in a potential *Schistosoma* vaccine trial. The community members were interested and willing to engage in discussion about a trial. However, for the success of such a trial, the concerns raised in this study need to be adequately addressed: the goals of the trial and requirements such as blood sample volume and trial duration need to be clearly explained, and adequate recognition must be given to participants’ contribution to the exercise.

As the world targets the elimination of neglected tropical diseases such as schistosomiasis, the perspectives of the target communities about the control strategies do provide very useful insights, especially to policy makers. Community-specific solutions can be designed to address potential barriers to the acceptability and sustainability of an otherwise scientifically proven intervention.

## Supporting information

S1 Text Approval letter—Uganda Virus Research Institute Research Ethics Committee. (PDF)

S2 Text Approval letter—Uganda National Council for Science and Technology. (PDF)

S3 Text Approval letter—London School of Hygiene and Tropical Medicine. (PDF)

S4 Text Topic guide—In-depth interviews. (DOCX)

S5 Text Topic guide—Key informant interviews. (DOCX)

S6 Text Topic guide—Focus group discussions. (DOCX)

S1 Table Thematic table–In-depth interviews. (XLS)

S2 Table Thematic table–Key informant interviews. (XLS)

S3 Table Thematic table–Focus group discussions. (XLSX)
