# Supplementary material for: Perceptions about interventions to control schistosomiasis among the Lake Victoria island communities of Koome, Uganda
Source: PLoS Negl Trop Dis. 2017 Oct 2;11(10):e0005982. doi: 10.1371/journal.pntd.0005982 (PMC5638603; doi:10.1371/journal.pntd.0005982)
Supplement: S1 Text — (PDF) [file pntd.0005982.s001.pdf]

UNHRO
Uganda National Health
Research Organisation

Uganda Virus Research Institute

Plot 51-59, Nakiwogo Road, Entebbe
P.O. Box 49, Entebbe-Uganda
Tel: +256 414 320 385 / 6
Fax: +256 414 320 483
Email: directoruvri@uvri.go.ug

REPUBLIC OF UGANDA

Our Ref: GC/127/15/05/510

26th May 2015

Your Ref: ……

Dr. Richard Edwin Sanya,

RE: UVRI REC review of protocol titled **"Community perceptions about interventions to control schistosomiasis in Koome sub-county, Mukono district Version 1.0, 7th April 2015."**

Thank you for submitting the above Protocol dated 10th April 2015 to UVRI Research Ethics Committee.

This is to inform you that your protocol was reviewed and met the requirements of the UVRI REC.

UVRI REC annual approval has been given for you to conduct your research up to 04th May 2016. Annual progress report and request for extension should be submitted to UVRI REC prior to the expiry date, to allow timely review.

The reviewed and approved documents included;

1. UVRI REC Application form
2. Study Proposal version 1.0, 7th April 2015
3. Consent forms
4. Applicant's CVs

You can now continue with your study after registration with the Uganda National Council for Science and Technology (UNCST).

Note: UVRI REC requires you to submit a copy of the UNCST approval letter for the above study before commencement.

Yours sincerely,

Anamale

Dr. Alice Namale
Vice Chair, UVRI REC
C.C The Director-UVRI,
Secretary, UVRI REC
