# Supplementary material for: Perceptions about interventions to control schistosomiasis among the Lake Victoria island communities of Koome, Uganda
Source: PLoS Negl Trop Dis. 2017 Oct 2;11(10):e0005982. doi: 10.1371/journal.pntd.0005982 (PMC5638603; doi:10.1371/journal.pntd.0005982)
Supplement: S2 Text — (PDF) [file pntd.0005982.s002.pdf]

Uganda National Council for Science and Technology

(Established by Act of Parliament of the Republic of Uganda)

UNCST

03/08/2015

Our Ref: SS 3831

Alison M. Elliott
MRC/UVRI Uganda Research Unit on AIDS
Uganda Virus Research Institute
Entebbe

Re: Research Approval: **Community perceptions about interventions to control schistosomiasis in Koome sub-county, Mukono district**

I am pleased to inform you that on **03/07/2015**, the Uganda National Council for Science and Technology (UNCST) approved the above referenced research project. The Approval of the research project is for the period of **03/07/2015** to **03/01/2016.**

Your research registration number with the UNCST is **SS 3831**. Please, cite this number in all your future correspondences with UNCST in respect of the above research project.

As Principal Investigator of the research project, you are responsible for fulfilling the following requirements of approval:

1. All co-investigators must be kept informed of the status of the research.
2. Changes, amendments, and addenda to the research protocol or the consent form (where applicable) must be submitted to the designated local Institutional Review Committee (IRC) or Lead Agency for re-review and approval **prior** to the activation of the changes. UNCST must be notified of the approved changes within five working days.
3. For clinical trials, all serious adverse events must be reported promptly to the designated local IRC for review with copies to the National Drug Authority.
4. Unanticipated problems involving risks to research subjects/participants or other must be reported promptly to the UNCST. New information that becomes available which could change the risk/benefit ratio must be submitted promptly for UNCST review.
5. Only approved study procedures are to be implemented. The UNCST may conduct impromptu audits of all study records.
6. A progress report must be submitted electronically to UNCST within four weeks after every 12 months. Failure to do so may result in termination of the research project.

Below is a list of documents approved with this application:

	**Document Title**	**Language**	**Version**	**Version Date**
1	Research Proposal	English	N/A	April 2015
2	Interview Guide	English /Luganda	2.0	May 2015
3	Consent Signature Sheet	English	2.0	May 2015

Yours sincerely,

Hellen N. Opolot
for: Executive Secretary
UGANDA NATIONAL COUNCIL FOR SCIENCE AND TECHNOLOGY

cc Chair, Uganda Virus Research Institute REC, Entebbe

LOCATION/CORRESPONDENCE

Plot 6 Kimera Road, Ntinda
P. O. Box 6884
KAMPALA, UGANDA

COMMUNICATION

TEL: (256) 414 705500
FAX: (256) 414-234579
EMAIL: info@uncst.go.ug
WEBSITE: http://www.uncst.go.ug
