# Supplementary material for: Perceptions about interventions to control schistosomiasis among the Lake Victoria island communities of Koome, Uganda
Source: PLoS Negl Trop Dis. 2017 Oct 2;11(10):e0005982. doi: 10.1371/journal.pntd.0005982 (PMC5638603; doi:10.1371/journal.pntd.0005982)
Supplement: S4 Text — (DOCX) [file pntd.0005982.s004.docx]

| **Community perceptions about interventions to control schistosomiasis in Koome sub-county, Mukono district** | |
| --- | --- |
| A LaVIISWA sub-study | |
| **GUIDE FOR IN-DEPTH INTERVIEWS** | |
| **First interview** | |
| 1 | Background information |
|  | Ask about the respondent’s background: age, gender, level of education, occupation, tribe , marital status, other household members in the family, how long the respondent has lived in Koome sub-county, housing, method of disposal of stool, any occupant in the household who is ill/sick  [Apologise if the participant has recently given the same information for the LaVIISWA surveys and explain that this data is being handled separately] |
| **Second interview** | |
| 2 | Assessing awareness of existence, causes, transmission, health problems and control of schistosomiasis/ Bilharzia |
|  | Assess the respondent’s knowledge by asking the following questions   - What are the most important health problems in your community? - Have you heard about a condition called Bilharzia or schistosomiasis? - Does Bilharzia exist in your community? - Are there local names for Bilharzia? - How bad do you think the problem of Bilharzia is, compared with other health issues in the community? - What do you think causes Bilharzia? - How is Bilharzia transmitted? - Who is at most risk of contracting Bilharzia? - Who is at most risk of developing serious problems from Bilharzia? - What are the symptoms and signs of Bilharzia? - How can you prevent and control Bilharzia? - Have you or any family member suffered from Bilharzia? If yes, where was treatment sought and at what cost? - How does Bilharzia affect a person’s day to day activities when infected? - What have you done to prevent and control Bilharzia? |
| 3 | Assessing attitudes towards the various strategies for controlling schistosomiasis/ Bilharzia |
|  | Ask the respondent about what he/she feels about the following methods for controlling Bilharzia and reasons. If necessary explain what is meant by each strategy.   - Mass administration of praziquantel - Disposal of faeces in toilets/ latrines - Provision and use of safe water supplies - Minimizing contact with infested water (the lake) - Biological control of snails |
| 4 | Determining the schistosomiasis/ Bilharzia control interventions most acceptable to the community |
|  | Ask the respondent the following questions   - How should the community provide interventions to control Bilharzia when the LaVIISWA-provided interventions end? - Which interventions do you think would work? - Which interventions are you willing to adopt? - Who should provide these interventions? - How can people be motivated to use these interventions? - What obstacles do you think will be faced when providing these interventions? |
| 5 | Determining the readiness of the community for *Schistosoma* vaccine trials |
|  | Explain to the participant that, at present, there are no vaccines for Bilharzia, but that researchers are trying to develop one. As part of the development process it will first be tested in laboratory animals, but will then need to be tested in people. First in small numbers to check that it is safe, and then in large numbers to find out whether it works. It will be important to test the vaccine among the communities where it would be used, if it is found to work.  Ask the respondent the following questions   - What do you know about vaccines and how they work? - What is your opinion about a vaccine for preventing Bilharzia?   Please imagine that you are asked to take part in a vaccine trial.   - Would you be willing to participate in a study to determine whether the vaccine works? It can be important to follow people up for quite a long time in a vaccine trial. Would you be willing to participate in a study for a duration of 3 years? - It is also important to test how someone responds to the vaccine several times immediately after it has been given. Sometimes (depending on the vaccine) a person may need to be given several doses (as is done in infants). Would you be available for study visits 3 days after every vaccination and for scheduled visits at 0,1,3,6 months from the start of the study? - Would you be willing to accept vaccine administration by injection? - When testing a vaccine, it is usual for some people to be given the real vaccine and some people to be given and injection that looks identical, but contains no active ingredients [this is called a placebo]. People are usually allocated to receive the real vaccine or the placebo by “randomization” – which means a process that is like a lottery. Would you be willing to take part, knowing that you might be given the placebo? - In order to test whether the vaccine is working it may be necessary to take larger blood samples than we have requested during LaVIISWA. Would you grant permission to have larger volumes (8–10 teaspoons) of blood drawn? - For women only: it is usual to ask women to avoid becoming pregnant while a new vaccine is being tested (because effects on an unborn baby may be uncertain). Would you agree to a request to delay or defer pregnancy for 10 months? |
