# Supplementary material for: Perceptions about interventions to control schistosomiasis among the Lake Victoria island communities of Koome, Uganda
Source: PLoS Negl Trop Dis. 2017 Oct 2;11(10):e0005982. doi: 10.1371/journal.pntd.0005982 (PMC5638603; doi:10.1371/journal.pntd.0005982)
Supplement: S6 Text — (DOCX) [file pntd.0005982.s006.docx]

| **Community perceptions about interventions to control schistosomiasis in Koome sub-county, Mukono district** | |
| --- | --- |
| A LaVIISWA sub-study | |
| **GUIDE FOR FOCUS GROUP DISCUSSIONS** | |
| Apologise if the participants have recently given the same information for the LaVIISWA surveys and explain that this data is being handled separately | |
| 1 | Assess awareness of existence, causes, transmission, health problems and control of schistosomiasis |
|  |  |
| 2 | Assess attitudes towards the various strategies for controlling schistosomiasis |
|  |  |
| 3 | Assess the schistosomiasis control interventions the community finds most acceptable |
|  |  |
| 4 | Assess whether the community is willing to participate in *Schistosoma* vaccine trials |
|  |  |
