# Supplementary material for: Perceptions about interventions to control schistosomiasis among the Lake Victoria island communities of Koome, Uganda
Source: PLoS Negl Trop Dis. 2017 Oct 2;11(10):e0005982. doi: 10.1371/journal.pntd.0005982 (PMC5638603; doi:10.1371/journal.pntd.0005982)
Supplement: S3 Text — (PDF) [file pntd.0005982.s003.pdf]

London School of Hygiene & Tropical Medicine
Keppel Street, London WC1E 7HT
United Kingdom
Switchboard: +44 (0)20 7636 8636

www.lshtm.ac.uk

Observational / Interventions Research Ethics Committee

Prof Alison Elliott
Professor
Department of Clinical Research (CRD)
LSHTM

23 June 2015 23 June 2015

Dear Prof Elliott ,

Study Title: Community perceptions about interventions to control schistosomiasis in Koome sub-county, Mukono district

LSHTM ethics ref: 10109

Thank you for your application for the above research, which has now been considered by the Observational Committee.

Confirmation of ethical opinion

On behalf of the Committee, I am pleased to confirm a favourable ethical opinion for the above research on the basis described in the application form, protocol and supporting documentation, subject to the conditions specified below.

Conditions of the favourable opinion

Approval is dependent on local ethical approval having been received, where relevant.

Approved documents

The final list of documents reviewed and approved by the Committee is as follows:

Document Type	File Name	Date	Version
Protocol / Proposal	LaVIISWA Social Sciences Study version 1	07/04/2015	1
Information Sheet	1. LaVIISWA social science substudy consent form_indepth interviews_English_v2_May 2015	11/05/2015	2
Information Sheet	1. LaVIISWA social science substudy consent form_indepth interviews_Luganda_v2_May 2015	11/05/2015	2
Information Sheet	2. LaVIISWA social science substudy consent form_key informant interviews_English_v2_May 2015	11/05/2015	2
Information Sheet	2. LaVIISWA social science substudy consent form_key informant interviews_Luganda_v2_May 2015	11/05/2015	2
Information Sheet	3. LaVIISWA social science substudy consent form_focus group discussions_English_v2_May 2015	11/05/2015	2
Information Sheet	3. LaVIISWA social science substudy consent form_focus group discussions_Luganda_v2_May 2015	11/05/2015	2
Local Approval	scan0354	26/05/2015	n/a
Investigator CV	CV, short, Alison Elliott	31/05/2015	n/a

After ethical review

The Chief Investigator (CI) or delegate is responsible for informing the ethics committee of any subsequent changes to the application. These must be submitted to the Committee for review using an Amendment form. Amendments must not be initiated before receipt of written favourable opinion from the committee.

The CI or delegate is also required to notify the ethics committee of any protocol violations and/or Suspected Unexpected Serious Adverse Reactions (SUSARs) which occur during the project by submitting a Serious Adverse Event form.

At the end of the study, the CI or delegate must notify the committee using an End of Study form.

All aforementioned forms are available on the ethics online applications website and can only be submitted to the committee via the website at: http://leo.lshtm.ac.uk

Additional information is available at: www.lshtm.ac.uk/ethics

Yours sincerely,

Professor John DH Porter
Chair

ethics@lshtm.ac.uk
http://www.lshtm.ac.uk/ethics/

Improving health worldwide
